# Supplementary material for: In Situ Synthesis of C-N@NiFe2O4@MXene/Ni Nanocomposites for Efficient Electromagnetic Wave Absorption at an Ultralow Thickness Level
Source: Molecules. 2022 Dec 27;28(1):233. doi: 10.3390/molecules28010233 (PMC9822367; doi:10.3390/molecules28010233)
Supplement: Supplementary file 1 [file molecules-28-00233-s001.zip › molecules-2094570-supplementary.pdf]

Supplementary Information

In Situ Synthesis of C-N@$NiFe_2O_4$@MXene/Ni Nanocomposites for Efficient Electromagnetic Wave Absorption at an Ultralow Thickness Level

Qing Sun [1,†], Xin Yang [1,†], Tie Shu [1], Xianfeng Yang [2], Min Qiao [1], Dashuang Wang [3], Zhaohui Liu [1,*], Xinghua Li [2], Jinsong Rao [3], Yuxin Zhang [3], Pingan Yang [4] and Kexin Yao [1,*]

1 Multi-scale Porous Materials Center, Institute of Advanced Interdisciplinary Studies, School of Chemistry and Chemical Engineering, Chongqing University, Chongqing 400044, China
2 State Key Laboratory of Photon-Technology in Western China Energy, School of Physics, Northwest University, Xi'an 710127, China
3 College of Material Science and Engineering, Chongqing University, Chongqing 400044, China
4 School of Automation, Chongqing University of Posts and Telecommunications, Chongqing 400065, China
* Correspondence: zhaohui.liu@cqu.edu.cn (Z.L.); kexinyao@cqu.edu.cn (K.Y.)
† These authors contributed equally to this work.

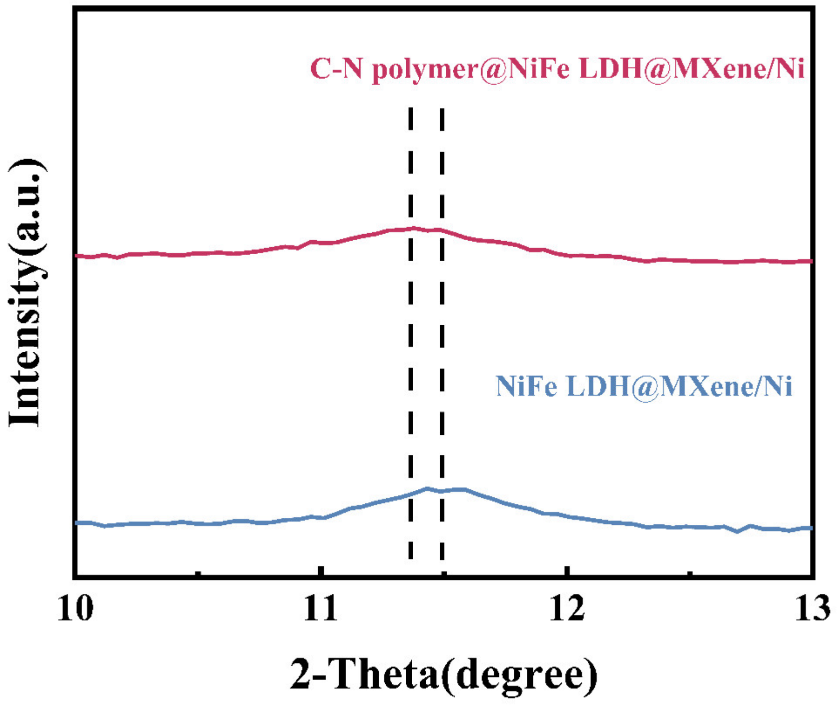

Figure S1. The partially enlarged detail of XRD patterns.

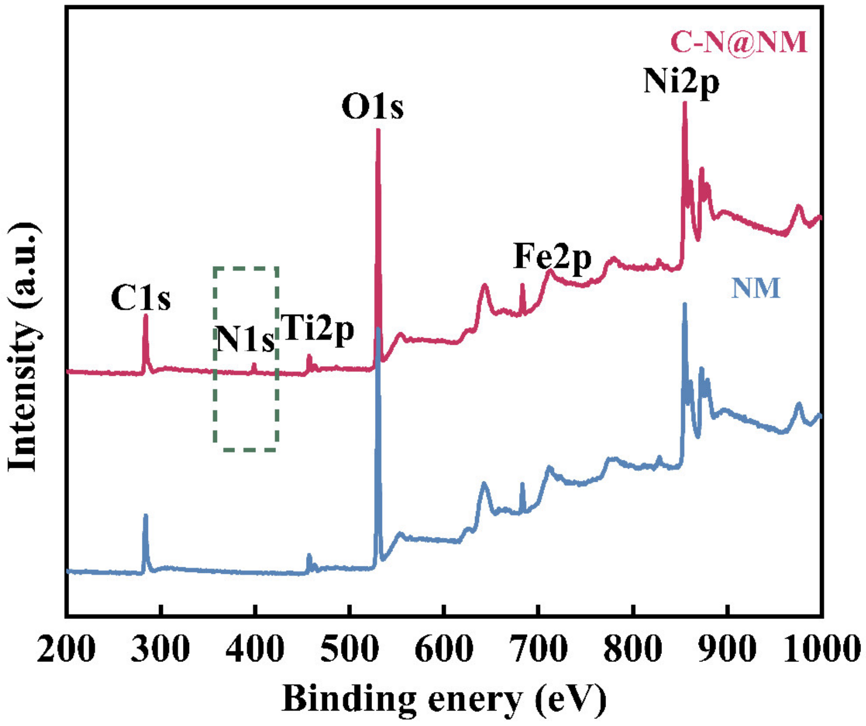

Figure S2. The wide-scan XPS spectrum of NM and C-N@NM.

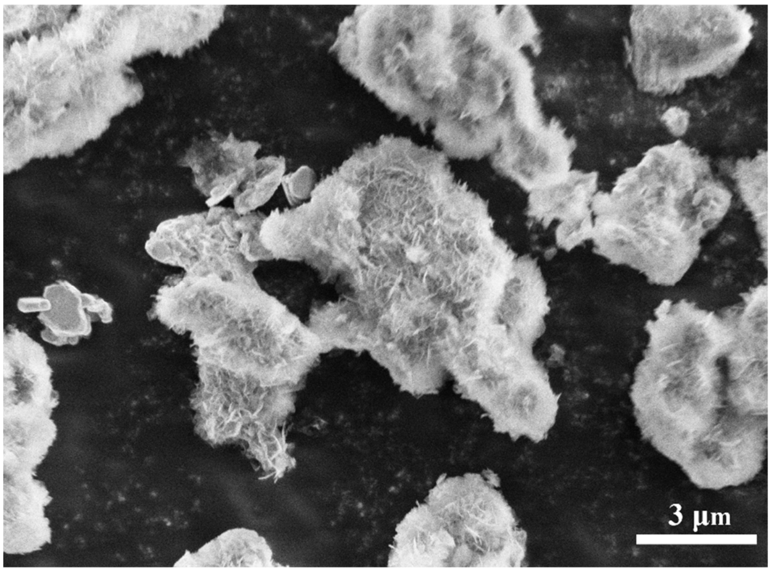

Figure S3. SEM image of NiFe LDH@MXene/Ni.

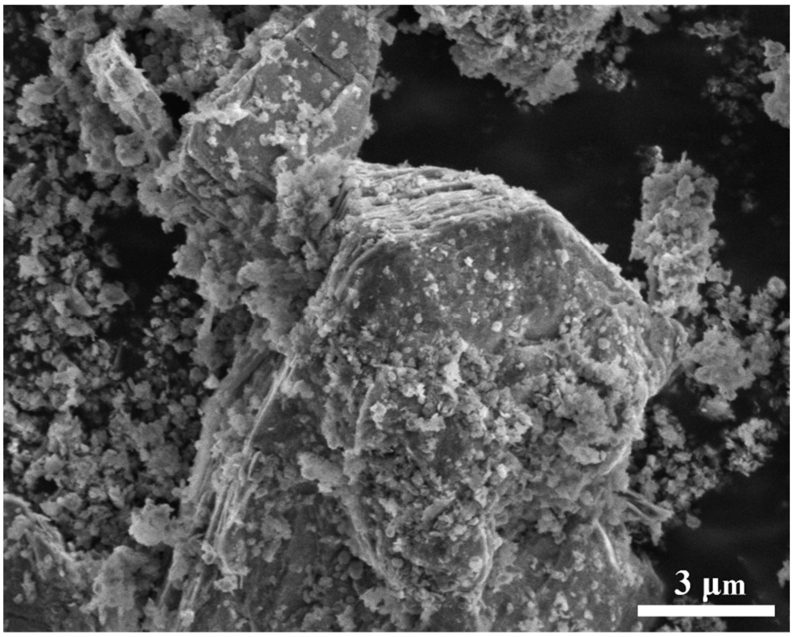

Figure S4. SEM image of NM.
